# Severe Lactic Acidosis Due to Acute Intoxication by Emtricitabine/Tenofovir Alafenamide

**DOI:** 10.7759/cureus.19008

**Published:** 2021-10-24

**Authors:** Swethapriya Chaparala, Rafael C Da Silva, John Paul Papadopoulos

**Affiliations:** 1 Internal Medicine, Piedmont Athens Regional Medical Center, Athens, USA

**Keywords:** toxicity, metabolic acidosis, lactic acidosis, intoxication, safety, emtricitabine, tenofovir, descovy

## Abstract

A 46-year-old female with a history of generalized anxiety disorder was admitted after intentional ingestion of an unknown amount of emtricitabine/tenofovir alafenamide (Descovy®) in a suicidal attempt. Patient was emergently intubated secondary to severe agitation and inability to protect airways. Patient developed severe lactic acidosis early in the admission, secondary as to a possible mitochondrial toxicity. Failed attempts to fluid resuscitation with Lactate Ringer®, eventually warranted to start the patient on norepinephrine infusion. Metabolic acidosis remained refractory to bicarbonate bolus and infusion. Hypothermia and hypoglycemia were corrected. Despite the initial approach, the patient remained acidotic, and the nephrology was consulted for emergent continuous renal replacement therapy (CRRT). After three days of intensive care unit stay and CRRT, the patient improved and was successfully decannulated. Her metabolic profile also showed remarkable improvement and the metabolic lactic acidosis resolved. The previous formulation of tenofovir with disoproxil fumarate is associated with severe lactic acidosis due to inhibition of mammalian mitochondrial DNA polymerase. Risk factors include liver cirrhosis, chronic kidney disease, hepatitis B and C coinfection, and metformin use. The new pharmaceutical formulation of tenofovir with alafenamide (TAF) has caused a significant decrease in the incidence of lactic acidosis. However, its real incidence and the usual plasma level to induce toxicity and mitochondrial dysfunction are unknown. The aim of this report is to highlight the risk of severe lactic acidosis with the use of TAF.

## Introduction

Nucleotide/nucleoside reverse transcriptase inhibitors (NRTI) are the cornerstone in the treatment of human deficiency virus (HIV) infection. Mitochondrial dysfunction is a well-known complication associated with NRTI, remarkably with stavudine (d4T) and didanosine (ddI) [1-4]. It manifests with acute liver failure and severe lactic acidosis [5-7]. Tenofovir disoproxil fumarate (TDF) was the first nucleotide reverse transcriptase inhibitor with activity against HIV introduced in the United States in 2001 [8]. It was reported that the combination of TDF and ddI results in an increase in the serum concentration of ddI, which can augment the risk of toxicity [9,10]. However, severe lactic acidosis rarely happens with the use of tenofovir, given its lower affinity to mitochondrial DNA polymerase gamma [4,11]. In 2015, another formulation of tenofovir with alafenamide (TAF) showed better safety and tolerability profile with lower impact on renal function as well as in bone mineral density without decreased efficacy. TAF is more stable in the plasma and offers higher intracellular concentration in the lymphocytes cell population [12-14]. With the advent of TAF, a component of Descovy®, the incidence of severe lactic acidosis and other renal dysfunctions has decreased [13]. The rationale of this case report is to highlight the risk of severe lactic acidosis due to acute intoxication of TAF/emtricitabine (Descovy®) and its management.

## Case presentation

This is a 46-year-old female with a history of generalized anxiety disorder who presented to the emergency department four hours after intentional ingestion of tenofovir alafenamide/emtricitabine (Descovy®). The patient had no formal diagnosis of HIV or hepatitis B. Medications were obtained from a close relative. She complained of severe nausea and lower back pain. Denied concurrent use of alcohol or salicylates. On presentation to the emergency department, the patient was agitated, tachypneic and hemodynamically stable. Lab work showed increased anion gap metabolic acidosis with acute kidney injury. Toxicology work-up for salicylate and alcohols was negative. A urine drug screen was positive for benzodiazepines and methamphetamine. Complete blood count was unremarkable and urinalysis negative for ketones. After 10 hours, the patient evolved to clinical and laboratorial deterioration consistent with hemodynamic instability, sinus tachycardia of 120 bpm, tachypnea at a respiratory rate of 50 bpm, hypothermia of 93.2˚ F and cyanosis. Blood pressure dropped to 80/60 mmHg. Follow up chemistry panel showed worsening of lactic acidosis and hypoglycemia (Table 1).

**Table 1:** Initial and follow up laboratory results. Pa02: arterial partial pressure of oxygen. Pvc02: central venous partial pressure of oxygen.

Laboratory	Initial results	Follow up results	Reference values
pH	7.04	< 6.77	7.35 – 7.45
Bicarbonate	11 mmol/L	4 mmol/L	22 – 28 mmol/L
Anion gap	24	39	10 – 12
Serum Creatinine	1.40 mg/dL	8 mg/dL	0.6 – 1.2 mg/dL
Lactic acid	10 mmol/L	27 mmol/L	< 2.3 mmol/L
Pa02		113 mm Hg	75 – 105 mm Hg
Pvc02		100 mm Hg	70 – 80 mm Hg

Patient was emergently intubated and transferred to the ICU. The patient was placed on a norepinephrine infusion following a failed resuscitation attempt with intravenous fluids. The metabolic acidosis remained refractory to bicarbonate bolus and drip. Hypothermia and hypoglycemia were corrected. Despite the initial approach, the patient remained acidotic, and the nephrology was consulted for emergent continuous renal replacement therapy (CRRT). After three days of ICU and CRRT, the patient improved and was successfully decannulated. Her metabolic profile also showed remarkable improvement and the metabolic lactic acidosis resolved. 

## Discussion

TAF has a better safety and tolerability profile compared to TDF. However, it can be associated with severe lactic acidosis as demonstrated by this case report. In 2018, Alsunaid et al. reported a 65-year-old male status post-hematopoietic stem cell transplant due to multiple myeloma, who received treatment for hepatitis B with TAF and died of severe lactic acidosis [15]. Our report is the first case associated with severe lactic acidosis due to intentional acute intoxication by TAF/emtricitabine (Descovy®) in an attempt at suicide. Given the clinical presentation of the patient with dramatic clinical worsening with cyanosis, hypoglycemia, and hypothermia, we believed patient presumably painted a picture of mitochondrial dysfunction. Tenofovir is a weak inhibitor of mammalian DNA polymerases that include mitochondrial DNA polymerase γ. Unfortunately, we were not able to precisely determine how much of the drug was ingested, neither the plasma concentration.

On the other hand, several studies demonstrated renal impairment with TDF including worsening creatinine clearance (CrCl) and proteinuria [13,14,16-19]. Interestingly, age older than 40 years was associated with a higher risk for renal abnormalities [20]. Additionally, concerns for permanent renal damage were suggested in patients taking highly active antiretroviral therapy (HAART) in this study published in 2009 [18]. Important to note, since 2003, several cases of fatal or severe lactic acidosis have been implicated with the use of TDF isolated [21-23] or in association especially with didanosine [9,24]. In addition, different associations including TDF and stavudine [5,24] as well as emtricitabine were also involved in episodes of lactic acidosis [25-27]. In two of those case reports, lactic acidosis was associated with metformin concomitantly with TDF. In one of them, continuous veno-venous hemofiltration was used successfully [25]. Lactic acid levels ranged between 5.3 to 14 mmol/l [24].

In 2015, TAF showed to be safer and better tolerated in comparison to TDF. To support this, no lactic acidosis was demonstrated by Triantos et al., who followed 107 patients with hepatitis B for five years [28]. Only in 2017, a case of acute tubular dysfunction due to mitochondrial toxicity was reported related to the use of TAF, however without severe lactic acidosis [29]. 

It is suggested that underlying conditions and combinations of drugs can precipitate lactic acidosis. This includes liver cirrhosis [21], coinfection with hepatitis C and B [21,23,26], diabetes mellitus [21,22,25], chronic kidney disease [11] and concurrent metformin use [24]. However, in 2012, Qayyum et al. reported a case of acute liver failure and severe lactic acidosis with TDF/FTC and efavirenz after three months of initiation of treatment in a 41-year-old male without previous liver disease [27]. Our patient had no comorbidities or risk factors that could precipitate lactic acidosis. 

The overall treatment reported is to stop the medication, follow lactic acid levels in the plasma, and start on bicarbonate drip. In cases of refractory metabolic acidosis, hemodialysis is frequently used. Hemodialysis in our patient was thought to play a major role in the elimination of medication with resolution of lactic acidosis. In the case reports listed, only four of the 14 patients survived after intoxication by tenofovir [5,9,11,15,16,21-25,27]. Our patient also had a favorable outcome without progression to chronic kidney disease. Likely, that was related to age and no comorbidities. Especially given the fatal nature of mitochondrial dysfunction, this warrants further studies.

## Conclusions

Severe lactic acidosis is a rare and severe complication of tenofovir, especially with tenofovir disoproxil fumarate ingestion. Apparently, there is high mortality associated with this condition. Possible risk factors include liver cirrhosis, hepatitis B and C, chronic kidney disease, and concomitant use of metformin. With the advent of tenofovir alafenamide, the risk of severe lactic acidosis decreased. However, it is still possible to develop severe lactic acidosis with this formulation, especially in the setting of acute intoxication. The management is based on the treatment of metabolic acidosis with bicarbonate drip and hemodialysis for refractory cases. Patients under TAF should be closely followed and it is suggested that evaluation for suicidal ideation should be considered, however, further studies might be necessary to support it.
